# *Eucalyptus* Oils Phytochemical Composition in Correlation with Their Newly Explored Anti-SARS-CoV-2 Potential: in Vitro and in Silico Approaches

**DOI:** 10.1007/s11130-024-01159-w

**Published:** 2024-03-16

**Authors:** Riham A. El-Shiekh, Mona M. Okba, Asmaa A. Mandour, Omnia Kutkat, Rana Elshimy, Hany A. Nagaty, Rehab M. Ashour

**Affiliations:** 1 Department of Pharmacognosy, Faculty of Pharmacy, Cairo University, Cairo, 11562 Egypt; 2 Pharmaceutical Chemistry Department, Faculty of Pharmacy, Future University in Egypt (FUE), Cairo, 11835 Egypt; 3 Center of Scientific Excellence for Influenza Viruses, National Research Centre, Giza, 12622 Egypt; 4 Department of Microbiology and Immunology, Egyptian Drug Authority, Cairo, Egypt; 5 Department of Microbiology and Immunology, Faculty of Pharmacy, Ahram Canadian University, Giza, Egypt; 6 School of Information Technology and Computer Science, Nile University, Giza, Egypt

**Keywords:** *Eucalyptus* essential oils, *Eucalyptus citriodora*, Pearson’s correlation, Globulol

## Abstract

Severe acute respiratory syndrome coronavirus 2 (SARS-CoV-2) is the latest arisen contagious respiratory pathogen related to the global outbreak of atypical pneumonia pandemic (COVID-19). The essential oils (EOs) of *Eucalyptus camaldulensis*, *E. ficifolia* F. Muell., *E. citriodora* Hook, *E. globulus* Labill, *E. sideroxylon* Cunn. ex Woolls, and *E. torquata* Luehm. were investigated for its antiviral activity against SARS-CoV-2. The EOs phytochemical composition was determined using GC/MS analysis. Correlation with the explored antiviral activity was also studied using multi-variate data analysis and Pearson’s correlation. The antiviral MTT and cytopathic effect inhibition assays revealed very potent and promising anti SARS-CoV-2 potential for *E. citriodora* EO (IC_50_ = 0.00019 µg/mL and SI = 26.27). The multivariate analysis revealed *α*-pinene, *α*-terpinyl acetate, globulol, γ -terpinene, and pinocarvone were the main biomarkers for *E. citriodora* oil. Pearson’s correlation revealed that globulol is the top positively correlated compound in *E. citriodora* oil to its newly explored potent anti SARS-CoV-2 potential. A molecular simulation was performed on globulol *via* docking in the main active sites of both SARS-CoV-2 viral main protease (Mpro) and spike protein (S). *In silico* predictive ADMET study was also developed to investigate the pharmacokinetic profile and predict globulol toxicity. The obtained *in silico*, in vitro and Pearson’s correlation results were aligned showing promising SARS-CoV-2 inhibitory activity of *E. citriodora* and globulol. This study is a first record for *E. citriodora* EO as a novel lead exhibiting potent in vitro, and *in silico* anti SARS-CoV-2 potential and suggesting its component globulol as a promising candidate for further extensive *in silico*, in vitro and in vivo anti-COVID studies.

**Supplementary Information:**

The online version contains supplementary material available at 10.1007/s11130-024-01159-w.

## Introduction

Essential oils (EOs) have a numerous biological activities; anti-parkinson, antifungal, anticarcinogenic, insecticide, anti-inflammatory, antimicrobial, antiviral and antioxidant [1, 2].The interest in EOs has been growing in food, cosmetic, medical, and pharmaceutical fields [3].

EOs and their pure compounds have been expressed as valuable antiviral drug candidates against RNA and DNA viruses in various host cell lines by blocking different steps of the viral life cycle [4]. These effects are due to the oil mono- and sesquiterpene hydrocarbons, alcoholic, phenolic, and other oxygenated constituents [4]. They have been documented against a variety of viruses; avian influenza A virus, human herpes virus, human immunodeficiency virus, Zika virus, and influenza A virus (H1N1) [5].

In this context, the biological activity of EOs could be extremely useful for management of the COVID-19 pandemic. Recently many plants EOs have been reported to exhibit a significant antiviral potential against SARS-CoV-2. *Allium sativum, Melissa officinalis*, *Citrus limon, Eugenia brasiliensis*, *Cedrus libani*, *Geranium dissectum, Zataria multiflora*, *Zingiber zerumbet* and, *Vetiveria zizanoides.* EOs together with their derivative compounds have been suggested as potent anti-COVID drugs, which could hinder CoV entry into the human body [6, 7].

The symptoms of SARS-CoV-2 have an incubation period of 2 weeks until the signs; cough, fever, lung damage, and dyspnea are expressed [8]. While studying drug targeting for the optimization of novel SARS-CoV-2 inhibitors, it was noted that the main protease (Mpro), as an ideal target for drug development, is responsible for major vital functions including the viral replication processes, with a highly conserved catalytic domain. Also, angiotensin-converting enzyme-2 (ACE2) receptor showed a crucial role in infection through a receptor-mediated interaction. Where a great similarity between SARS-CoV-2 and SARS virus was computationally proved, with the highest affinity to receptor ACE2 as a first binding step at the cell membrane. Followed by SARS-CoV-2 entry being mediated by spike glycoprotein. This allows the virus entry into the host cell through the active receptor-binding domain (RBD) in the spike protein (S) found on surface of the virus [9]. Recently it was documented that the blocking ACE2 and the (S) is one possible way of inhibiting the entrance of SARS-CoV-2 to the host cell. Thus, drugs that can inhibit one of these proteins would be identified as potential new anti-COVID compounds [9, 10].

*Eucalyptus* is a well-known genus belongs to family Myrtaceae. It is traditionally used to treat many disorders [11]. The *Eucalyptus* genus is characterized by 900 species and subspecies which is native to Australia and cultivated worldwide. This plants contains wide array of phytochemicals as essential oils, phloroglucinols, tannins, flavonoids, oleuropeic acid derivatives and triterpenes [12]. *Eucalyptus* possesses antiviral, antibacterial, anticancer, antioxidant, antiseptic, antigout and anti-inflammatory potentials [11, 13].

In a continuation of our previous study for exploring anti SARS-CoV-2 drugs [14] and our previous research on *Eucalyptus* antiviral potential [12], *Eucalyptus* EOs were selected for this study. *Eucalyptus* trees have perennial leaves that are odorous due to the presence of EOs, produced and stored in secretory cells. These EOs are aromatic, spicy, and colorless or pale yellow, or brownish or greenish in color [15]. *Eucalyptus* EOs are rich in monoterpenes and sometimes in sesquiterpenes. They are rich in 1,8-cineole, citral, citronellal and geranyl acetate [16]. *Eucalyptus* EOs exhibit potent antimicrobial, acaricidal, insecticidal, anticancer, and herbicidal properties. Their pharmaceutical industrial, and agrochemical applications in addition to food preservation properties are well documented [17].

There are few data about the potential of *Eucalyptus* EOs and its components against the COVID-19 virus. The present study aimed at evaluating the in vitro anti SARS-CoV-2 potential of six *Eucalyptus* species; *Eucalyptus camaldulensis* Dehnh, *E. ficifolia* F.Muell., *E. citriodora* Hook, *E. globulus* Labill, *E. torquata* Luehm., and *E. sideroxylon* Cunn. ex Woolls, and to correlate the observed in vitro activity if any with the phytochemical components of oils in an attempt to explore novel lead for management of COVID infection.

## Materials and Methods

The detailed procedures are described in the supplementary online resource.

## Results and Discussion

The current crisis caused by SARS-CoV-2 reveals the need for new potent antiviral drugs. Recent studies focused on exploring new compounds from medicinal plants EOs as a promising sources exhibiting potential against virus infection and replication [18]. The antiviral activities of EOs against SARS-CoV-2 using several approaches such as in vitro, docking models or in clinical instances were previously investigated [19].

EOs lipophilic structure allows them to invade the virus envelope then, modulates the viral lipid double layer fluidity permitting the disturbance its life cycle [20]. EOs different antiviral mechanisms have been studied. Some exhibited their activity through immediate effects on free viruses, blocking of different virus life cycle steps for examples as fusion, replication and release from host cells, and inhibition of the key viral enzymes [5]. Some EO components were reported to diminish viral infectivity by more than 80% [21].

Eucalyptus EOs and some of their components were reported before for their antiviral properties against many viruses [21–24], but they were not tested before against the COVID-19 virus.

### Anti-SARS-CoV-2

The results were determined by calculating the CC_50_ in addition to the IC_50_ that is required to reduce the virus-induced cytopathic effect (CPE) by 50% (Fig. S1). The overall therapeutic activity of the active one was established in terms of the SI (the ratio of the CC_50_ to IC_50)_. The most promising one is *E. citriodora* with IC_50_ = 0.00019 µg/mL from the tested samples against SARS-CoV-2. High SI values (more than 10) recommend the drug is a good candidate as antiviral agent and that its effect is more directed toward the viral inhibition with less cytotoxic activity on the host cell [25]. This revealed a promising activity for *E. citriodora* leaves EO against SARS-CoV-2 with a significant SI (26.27 × 1000). *E. torquata* had high cytotoxicity effect on cells and so no further biological evaluation was carried out on *E. torquata* EO.

### GC/MS Analysis

The hydro distilled EOs obtained from six *Eucalyptus* species offered a range yield of 0.7–2.3% *v/w*. A total of 53 components, comprising 92.1-97.96% of the total composition of the different samples, were identified and quantified using GC-MS analysis. The chemical names and the area % of identified constituents are tabulated in Table S1. The results revealed that monoterpenes are the principal components of the tested oils, with a good percentage of sesquiterpenes constituents. Oxygenated monoterpenes are the most abundant phytoconstituents of *E. ficifolia* and *E. sideroxylon* oils amounted to 37.70% and59.41% of the identified compounds. On the other hand, the monoterpene hydrocarbon is the most abundant group in *E. citriodora* (64.2%) and *E. globulus* (39.8%) oils. *E. camaldulensis* (41.6%) and *E. torquata* (45.68%) oils are the richest oils in oxygenated sesquiterpenes (Table S1).

In this study, *E. citriodora* showed very promising anti SARS-CoV-2 potential. GC/MS of the EOs from six *Eucalyptus* leaves showed variation in their essential constituents. Monoterpenes, oxygenated monoterpenes, and sesquiterpenes were the major class of essential constituents. Many of the GC-MS detected components of the EOs under investigation are previously reported to exhibit antiviral activity. *β*-pinene, *α*-pinene, *γ*-terpinene, 1,8-cineole and terpinen-4-ol revealed anti-herpes simplex virus type 1 activity more than 80% [21]. α-terpinyl acetate was reported to play a key role in inhibiting replication of SARS-CoV-2 via binding to Nsp15 viral protein and they might [26]. Eucalyptol (1,8 cineole) was previously reported as a promising therapeutic agent in the management of COVID-19 [27]. It was well documented that α-pinene not only exerted potent antiviral activity against HSV-1 [28] but also both α and *β*-pinene were reported to inhibit the binding between RNA and the IBV N-protein, consequently, possess anti-IBV activity [29]. Furthermore, *in silico* studies highlighted their ability of binding to the active site of human serine protease TMPRSS2, Mpro, and spike (S) glycoprotein leading to their anti-SARS-CoV-2 potential [4, 28]. *α* and *β*-Pinene were reported in several recent *in silico* studies to exhibit anti-COVID potential [18]. Also, borneol exhibited a potent antiviral activity against orthopoxvirus and influenza A virus [30].

### Multivariate Data Analysis and Pearson’s Correlation

Although some differences in the six species phytochemical pattern were noticed by simple visual inspection of GC/MS results (Table S1), MVDA including hierarchical cluster analysis (HCA) and principal component analysis (PCA) were used to evaluate the chemical diversity and explore the relative variability within the oils GC-MS derived dataset in a more holistic way. PCA revealed that the six species were divided into 3 main clusters: the first cluster representing *E. citriodora* oil only, the second cluster representing *E. camaldulensis*, *E. ficifolia*, and *E. globulus* oils, the third cluster including *E. sideroxylon* and *E. torquata* oils (Fig. S2A). This confirms that *E. citriodora* oil was different in the levels and occurrence of certain phytochemicals and discriminated from the other five *Eucalyptus* species. Two principal components cumulatively accounted for 56.97% of the total variance (Fig. S2A). PCA revealed that PC1 and PC2 explained 21.94% and 35.03% of the variance respectively.

The PCA of the main discriminatory markers of each cluster (Fig. S2B) revealed that *α*-pinene, *α*-terpinyl acetate, globulol, *γ*-terpinene, borneol, *β*-pinene, epiglobulol, *γ*-terpinyl acetate, fenchol, campholenal, piperitone, citronellyl acetate, trans-carveol, and pinocarvone were the main biomarkers for *E. citriodora* oil while linalool, *m*-cumenol, and terinen-4-ol are the main discriminatory biomarkers for the second cluster *E. camaldulensis*, *E. ficifolia*, and *E. globulus* oils.

The dendrogram (Fig. S3) of the six *Eucalyptus* species were grouped according to their chemical profiles into four clusters (clusters I-IV). The first cluster (cluster I) was composed of *E. citriodora* oil only which was more diverged among all tested species. The second cluster (cluster II) comprised *E. camaldulensis* oil. The third and fourth clusters comprised; cluster III: *E. sideroxylon* and *E. torquata* oils and cluster IV: *E. ficifolia*, and *E. globulus* oils, suggesting that they have comparable phytochemical profiles.

Quantitative heatmap (Fig. S3) was constructed to visualize and assess qualitative and quantitative differences across the tested eucalyptus oils. The heatmap revealed the main clusters of phytochemicals of highest abundance in each of the six studied *Eucalyptus* species. From the color code (Fig. S3) compounds with red colors are those of the highest abundance. *α*-pinene, *α*-terpinyl acetate, (-)-globulol, *γ*-terpinene, borneol (2.48%), *β*-pinene, epiglobulol, *γ*-terpinyl acetate, fenchol (0.83%), campholenal (0.44%), piperitone, citronellyl acetate, trans-carveol, pinocarvone are of highest abundance in *E. citriodora* oil relative to other eucalyptus oils. On the other hand, *α*-ylangene, cis sabinene hydrate, spathulenol, cis thujopsene, isospanthlenol, *α*-cadinol, *β*-myrecene, Selina 5, 11 diene, delta 2 carene, isothujyl acetate were the main phytoconstituents discriminating *E. camaldulensis* oil. *E. sideroxylon* oil was distinguished by the relative high abundance of α- phellandrene, trans thujopsene, 1,8- cineole, and viridiflorol. *α-*Thujene, trans-sabinol, γ-eudesmol, and torquatone were detected as the majors phytoconstituents discriminating *E. torquata* oil. Trans nerolidol, *p* cymene7ol, citronellal, isoborneol were of highest relative abundance in *E. ficifolia* oil. Finally, cis sabinene hydrate acetate, carvacrol, and estragol are the characteristic components of *E. globulus* oil.

Although some differences in the six species essential oil chemical pattern were observed by simple visual inspection of the PCA (Fig. S2), and the heatmap (Fig. S3) applied on the identified compounds in each tested oil, it was crucial to apply Pearson’s linear correlation to explore whether certain essential oil component is significantly correlated with the very potent newly observed *E. citridora* oil anti-SARS-CoV-2 potential or this potent activity was due to the synergistic activity of many components at the same time. Hence, *E. torquata* oil had high cytotoxic effect, the GC/MS results of this oil were not included in Pearson’s correlation.

Pearson’s linear correlation results revealed that globulol was the top positively correlated compound in the *E. citriodora* oil (Fig. S4A). Chemometric study was performed based on GC data where PCA revealed the biomarkers of each EO sample and discriminated them into separated clusters. Globulol is an antimicrobial sesquiterpene alcohol previously isolated from the fruits of *E. globulus* Labill (Myrtaceae) [31]. It has also been reported to have potent antioxidant and antifungal activities [32]. On the other hand, carvacrol, estragole, cis-sabinene hydrate acetate, trans*-p*-menth-1-en-3-ol, *α*-thujene, *β*-cymene, trans-sabinene hydrate, *m*-cumenol, cryptone, cuminaldehyde, and *β*-phellandrene were negatively correlated to the observed *E. citriodora* oil. Structures of the top ten positively correlated compounds were illustrated in Fig. S4B. Hence the correlation coefficient of globulol is 0.5 and since many components of *E. citriodora* oil *α*-pinene, *β*-pinene, *α*-terpinyl acetate, and 1,8 cineol (eucalyptol) are of well documented anti-viral and anti-COVID potential [18, 28, 29, 33], it was concluded that this very potent anti-COVID activity exhibited by *E. citriodora* oil was due to strong synergistic activity of several compounds with each other. In other words, the presence of *α*-pinene, *β*-pinene, α-terpinyl acetate, 1,8 cineol, globulol with each other was responsible for the activity and not to globulol only.

### Molecular Modeling

An *in-silico* docking study applying C-Docker protocol using Discovery Studio 4.0 Software on globulol was performed. After preparing the tested ligand, docking into each of the two binding sites of Mpro and S receptors was achieved using C-Docker protocol. The analysis study was further applied to distinguish the binding mode of globulol and to interpret the estimated biological results. The best pose of globulol showing comparable binding mode (with the key amino acids in the binding site) to the complexed ligand was selected. Also, validation was confirmed by re-docking of the complexed ligands in the specific active site of each of the two targets showing RMSD value = 0.5 A° as good validation results.

The main reported binding interaction at the active sites from the previous literature and the docking results of the ligand as well were used to evaluate the docking results of globulol. C-Docker results on Mpro active site (E=-26.19 Kcal/mol) in Fig. S5, showed that it retained the essential hydrogen bond interaction with GLU166 as in boceprevir ligand (E=-71.5 Kcal/mol). Besides the other reported hydrophobic interactions with HIS41, MET49 and MET165. Also, an interaction with CYS145 was recorded but with hydrophobic binding not hydrogen bond compared to ligand [34].

While docking results of globulol on spike (S) receptor was compared to NAG docking results besides the previously published essential interactions at the binding site [34]. It was observed that NAG (E=-24.12 Kcal/mol) showed one hydrogen bond HBD with GLY339 residue, this was replaced by two hydrogen bonds (1HBD and 1HBA) with ASN343 residue by globulol (E=-18.04 Kcal/mol) (Fig. S6), that was thought to enhance the binding affinity of globulol [34]. Besides, the hydrophobic interactions with PHE342 and LEU368 residues.

It’s worth noting that the glycosylated nature of spike proteins in Coronavirus with N-acetyl glucosamine (NAG) as the main component, influenced its structural packaging. And removal of glycosylation will not only distort but also affect its structure for proper binding [35].

Results of globulol docking on the two mentioned active sites showed promising estimated activity of the test compound as COVID-19 treatment candidate with good interaction with the essential amino acids in the binding sites (Fig. S7).

Also, an ADMET study was conducted to evaluate the pharmacokinetic profile of globulol using Discovery Studio 4.0 Software. The aim of the study was to investigate the drug-likeness of the compound based on correlating the chemical structure to several calculated parameters including; Aqueous solubility level, Atom based Log P98 (A LogP 98), Blood Brain Barrier Level, Cytochrome P450 2D6 (CYP2D6), Hepatotoxicity Probability, Absorption level, Plasma protein binding level (PPB Level), 2D polar surface area (ADMET 2D PSA) and toxicity being predicted by Komputer Assisted Technology (TOPKAT) [36].

Table S2 demonstrated the ADMET results showing that globulol exerts BBB level of 1, which indicates moderate BBB penetration. Absorption level value of 0, indicating good level of human intestinal absorption. With low ADME aqueous solubility level of 2. Log P parameter indicates the lipophilicity of the compound, it can be together with the polar surface area (PSA), used for evaluation. Where compounds that do not exceed the value of 5 (3.202 for globulol), are usually considered for their well absorption. On the other hand, the bioavailability is greatly related to the (PSA) property of the drug. As molecules with PSA < 140 (20.815 for globulol) show more passive absorption. Hereby, globulol showed hepatotoxicity probability. It is considered as Cytochrome P450 2D6 (CYP2D6) non inhibitor, with a probability for plasma protein binding. While toxicity prediction (TOPKAT) showed non mutagen result.

The *in-silico* ADMET prediction study of globulol showed good pharmacokinetic profile as a non CYP450 inhibitor with good intestinal absorption level showing good passive oral absorption and the most important is being a non-mutagen drug regarding toxicity prediction (Fig. S8).

Hence, globulol is the most correlated compound to the anti SARS activity with appropriate binding to the key amino acids, this suggested that *E. citriodora* oil, and globulol needs extensive in depth detailed in vitro, *in silico*, and in vivo studies to find out the possibility of their use in management of SARS-CoV-2 infection. Mechanism of antiviral studies on *E. citriodora* oil and globulol are highly recommended. In addition, EOs chemical composition may vary from one season to another even if extracted from the same plant, which can affect the biological response. Thus, it is highly recommended that further studies on seasonal variations of the studied species EOs composition to be conducted.

Consequently, this study reveals that *Eucalyptus* EOs have antiviral potential matching with the genus reported folk uses. Furthermore, our study recommended *E. citriodora* and its component globulol as antiviral agent against the pandemic COVID-19. The use of the EOs as natural antiviral drugs requests further detailed studies to approve their effectiveness and safety.

## Conclusion

Together with the approved vaccines, the innovation of prospective anti-COVID-19 drugs has been targeted. Our study revealed *E. citriodora* EO as a very potent anti SARS-CoV-2 potential. Multivariate analysis revealed *α*-pinene, *α*-terpinyl acetate, globulol, *γ*-terpinene, borneol, *β*-pinene, along with other components as the main *E. citriodora* EO biomarkers. Globulol was the top positively correlated compound with this newly explored activity. Molecular modeling revealed that globulol exhibited comparable results to the downloaded ligands at the active sites and confirmed the binding to the key amino acids with appropriate binding energy. This is the sole map representing the chemical profiling of some common and rare *Eucalytpus* species in correlation with their anti-SARS-CoV-2 potential. *E. citriodora* oil and globulol may serve as a potential source of plant-based drugs for SARS-CoV-2 control.

### Electronic Supplementary Material

Below is the link to the electronic supplementary material.

Supplementary Material 1

## Data Availability

No datasets were generated or analysed during the current study.
