# Supplementary material for: Eucalyptus Oils Phytochemical Composition in Correlation with Their Newly Explored Anti-SARS-CoV-2 Potential: in Vitro and in Silico Approaches
Source: Plant Foods Hum Nutr. 2024 Mar 16;79(2):410–6. doi: 10.1007/s11130-024-01159-w (PMC11178612; doi:10.1007/s11130-024-01159-w)
Supplement: Supplementary file 1 — Supplementary Material 1 [file 11130_2024_1159_MOESM1_ESM.docx]

**Material and Methods**

**Plant Material**

*Eucalyptus sideroxylon* Cunn. ex Woolls (No. 05.06.19 I) and *Eucalyptus torquata* [Luehm.](https://en.wikipedia.org/wiki/Luehm.) (No. 05.06.19 II) leaves were collected in May 2022 from El-Kobba palace, Cairo, Egypt. *Eucalyptus globulus* Labill (No. 05.05.2022 I) leaves were collected from the Experimental Station of Faculty of Pharmacy, Cairo University. *Eucalyptus camaldulensis* Dehnh, *E. ficifolia* [F.Muell.](https://en.wikipedia.org/wiki/F.Muell.), and *Eucalyptus citriodora* Hook (No. 05.05.2022 II-IV) leaves were collected from Giza Zoo, Giza, Egypt. The plants were kindly identified by Mrs. Therese Labib, Consultant of Plant Taxonomy at Ministry of Agriculture and the Former Director of El-Orman Botanical Garden. Voucher specimens were deposited in the museum of Pharmacognosy Department, Faculty of Pharmacy, Cairo University. Collection of the plant material is in accordance with institutional, national and international guidelines.

**Preparation of the Eucalyptus oils**

One kilogram of fresh leaves from each species was separately hydrodistilled until no more essential oil was obtained using a Clevenger apparatus. Oils were dried over anhydrous sodium sulphate. The oils were kept at -4^o^C in sealed vials for further biological and chemical analysis. The % yield was calculated on fresh weight basis (*v/w*); 0.7, 0.9, 1.1, 2.3, 1.5, and 1.8, respectively for *E. camaldulensis*, *E. ficifolia*, *E. citriodora*, *E. globulus*, *E. sideroxylon*, and *E. torquata*.

**MTT cytotoxicity assay**

The assay was performed according to Mosmann, *et. al.*, 1983 [1]. The details of the assay are in the supplementary file.

**Inhibitory concentration 50 (IC_50_) determination**

IC_50_ determination was carried out according to Kandeil, *et. al.*, 2021 [2]. The method details are mentioned in the supplementary file.

*Calculation of Selectivity Index*

The selectivity index (SI) is the ratio of the cytotoxic concentration of a sample to its effective concentration. The higher the SI ratio, the more efficient and safer a drug is. The SI value was calculated using the following equation: SI = CC_50_/IC_50_.

**GC/MS analysis**

GC/MS analysis was performed at the Faculty of Pharmacy, Ain Shams University (Cairo, Egypt) using Shimadzu GCMS-QP2010 (Koyoto, Japan) system connected to fused bonded column, Rtx-5MS, (Restek, USA) coupled to SSQ 7000, a quadruple mass spectrometer (Thermo-Finnigan, Bremen, Germany). The elution starts with isothermal temperature at 45 °C for 2 min, followed by programmed elution at 300 °C at 5 °C/min. The flow rate of helium was 1.41 mL/min [3]. The oil constituents were deconvoluted by using AMDIS software (www.amdis.net). The detected compounds were identified *via* comparing their mass spectra and retention indices (RI) to that reported in the Pherobase database and the NIST library. Final confirmation of identification was done using literature.

**Multivariate data analyses**

Multivariate data analysis (MVDA) was performed using FactoMineR, an R package for multivariate analysis [4, 5]. Principal Component Analysis (PCA) was applied on the GC/MS results to produce the graph of individuals (Eucalyptus oil of different species) and the graph of variables (identified compounds).

The heatmap was produced using R [5] to illustrate the concentration of the compounds in each species. The dendrogram illustrates the similarity within the compounds and within the species.

Pearson’s correlation was applied on the results of GC/MS analysis of the studied essential oils to explore the linear correlation between the different identified compounds and the newly observed anti COVID potential.

**Molecular Modeling**

The crystal structure of the two viral SARS-CoV-2 target proteins named main protease Mpro complexed with boceprevir ligand (PDB ID: 6XQU) and spike receptor binding domain (S) with ACE2 (PDB ID: 6M0J) were successfully downloaded from PDB (Protein Data Bank)[6]. Discovery Studio 4.0 Software was used to perform the molecular docking study. Clean Protein was completed to both proteins, and hydrogen atoms were added to amino acid residues to complete any missing residues. Also, unneeded water molecules were removed. Then, Force Field using CHARMm and partial charge MMFF94 were applied. Proteins were both prepared and minimized, and the active sites were well defined with grid extension value of 8. Both ligands: Boceprevir and 2-acetamido-2-deoxy-beta-D-glucopyranose (NAG) were removed prior to docking of the tested compound using C-Docker docking algorithm. Prepare Ligand was used to prepare Globulol using Rule Based ionization method.

Also, predictive ADMET study was applied via Discovery Studio 4.0 Software to investigate the pharmacokinetic properties including absorption, distribution, metabolism, and excretion (ADME), and determine the toxicity profile of globulol that would greatly influence the clinical impact of the drug molecule.

**MTT cytotoxicity assay[7]**

To assess CC_50_, stock solutions of the tested oils were prepared in 10 % DMSO in ddH_2_O and then diluted with DMEM to the working solutions. The oils cytotoxic activity was tested in VERO-E6 cells by using the MTT method with minor modifications. Briefly, the cells were seeded in 96 well-plates and incubated at 37 °C for 24 h in 5% CO_2_. Cells were treated with various concentrations of the tested compounds in triplicates (after 24 h). The supernatant was discarded, and cell monolayers were washed with sterile 1x PBS 3 times and MTT solution (20 µl of 5 mg/mL stock solution) was add and incubated for 4 h at 37 °C. In each well, the formed formazan crystals were dissolved with acidified isopropanol composed of 0.04 M HCl in absolute isopropanol = 0.073 mL hydrochloric acid in 50 mL isopropanol. Absorbance was measured at λ max 540 nm with 620 nm as a reference λ using a multi-well plate reader. The cytotoxicity % compared to the untreated cells was determined according to the following equation.

The plot of % Cytotoxicity versus tested sample concentrations was used to calculate the conc. which exhibited CC_50_ (50% cytotoxicity).

$\% Cytotoxicity = ((Abs.of cells without treatment-Abs. of cells with treatment) /(Abs. of cells without treatment ) X 100).$

**Inhibitory concentration 50 (IC_50_) determination [2]**

In 96-well tissue culture plates, 2.4×10^4^ Vero-E6 cells were distributed in each well and incubated overnight under 5% CO_2_ at a humidified 37°C condition. The cell monolayers were washed once with 1x phosphate buffer saline and then subjected to virus adsorption (hCoV-19/Egypt/NRC-03/2020 (Accession Number on GSAID: EPI_ISL_430820)) at room temperature for 1 h. The cell monolayers were overlaid with DMEM (100μl) containing varying concentrations of the test samples. After the incubation in 5% CO_2_ at 37°C for 72 h, the cells were fixed with 4% paraformaldehyde (100 μL) for 20 min then stained with crystal violet (0.1%) in distilled water at room temp for 15 min. The crystal violet dye was after that dissolved using 100 μL absolute methanol per well and the optical density of the color is measured at 570 nm using Anthos Zenyth 200rt plate reader (Anthos Labtec Instruments, Heerhugowaard, Netherlands). The IC_50_ of the tested sample is the concentration required to decrease the virus-induced cytopathic effect by 50%, relative to virus control.

**Results and Discussion**

***
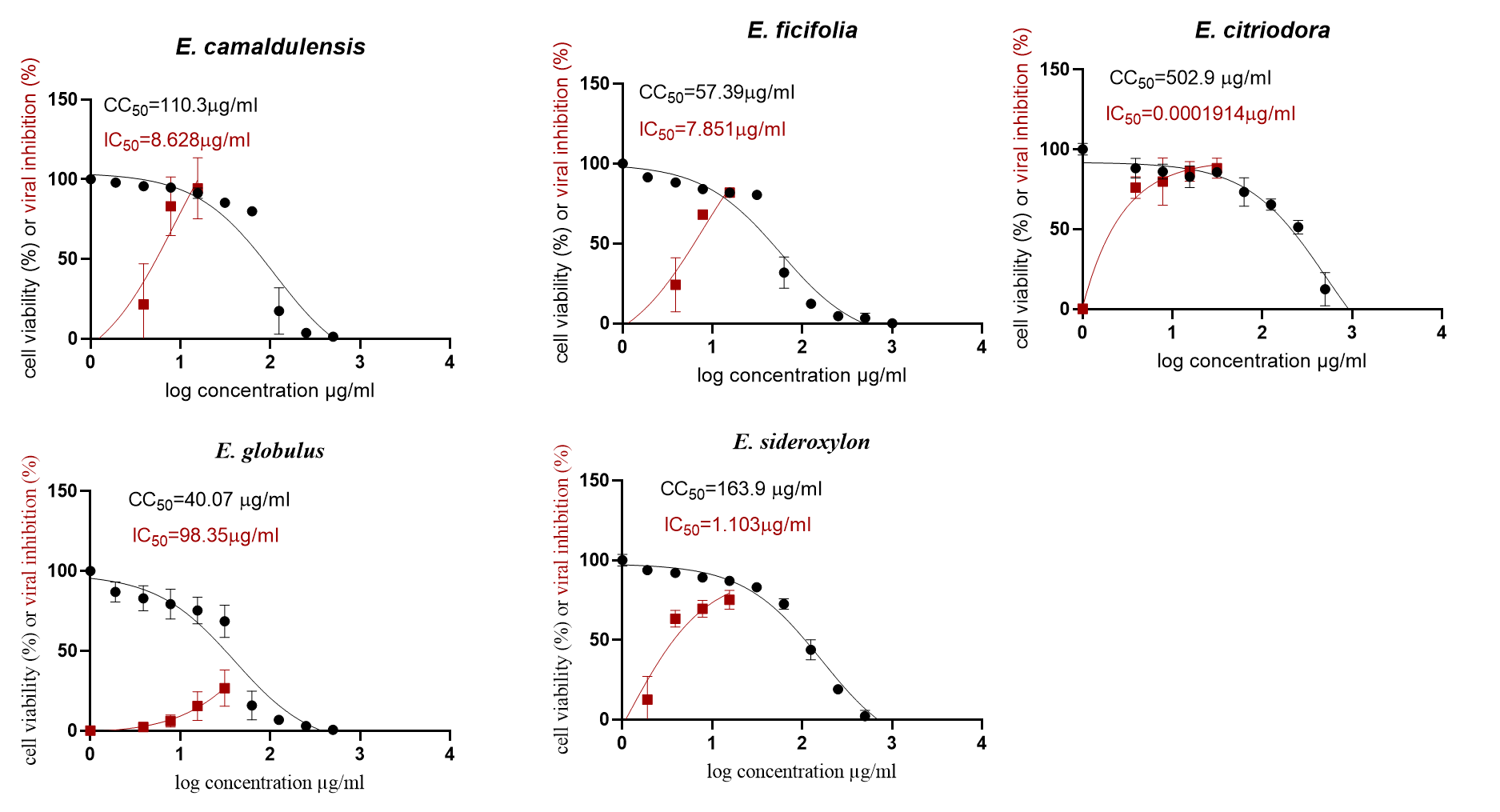
***

**Figure S1. Graph of the cytotoxic concentration 50 (CC_50_) and inhibitory concentration 50 (IC_50_) of the tested *Eucalyptus* oils.**

**Table S1. Identified metabolites of *Eucalyptus* oils using GC/MS analysis.**

| **Identified**  **Compounds** | **Kovat index** | | **Mol.**  **formula** | | ***E. camaldulensis*** | | ***E. ficifolia*** | | ***E. citriodora*** | | ***E. globulus*** | | ***E. torquata*** | | ***E. sideroxylon*** | | **Molecular weight** | |  |
| --- | --- | --- | --- | --- | --- | --- | --- | --- | --- | --- | --- | --- | --- | --- | --- | --- | --- | --- | --- |
| alpha-Thujene | 924 | | C_10_H_16_ | | 0.76±0 | | 1.27±0.03 | | 0.84±0.02 | | 1.5±0.01 | | 19.8±0.02 | | 1.14±0.03 | | 136 | |  |
| alpha-Pinene | 932 | | C_10_H_16_ | | 0.81±0.01 | | 8.03±1.79 | | **53.3±0.59** | | 0.98±0.01 | | 0.57±0.001 | | 2.7±0.05 | | 136 | |  |
| beta-Pinene | 974 | | C_10_H_16_ | | 0.25±0 | | 0.5±0.01 | | 1.49±0 | | 0.39±0 | |  | | 0.38±0.02 | | 136 | |  |
| beta-Myrecene | 988 | | C_10_H_16_ | | 0.2±0.01 | |  | |  | |  | |  | |  | | 136 | |  |
| delta-2-Carene | 1001 | | C_10_H_16_ | | 0.28±0.03 | |  | |  | |  | |  | |  | | 136 | |  |
| alpha-Phellandrene | 1002 | | C_10_H_16_ | | 0.29±0 | | 1.88±0.03 | | 0.71±0.11 | | 1.22±0.02 | |  | | 10.6±0.11 | | 136 | |  |
| delta-4-Carene | 1008 | | C_10_H_16_ | |  | | 0.14±0.02 | | 0.18±0 | | 0.16±0.03 | |  | | 0.14±0.01 | | 136 | |  |
| para-Cymene | 1020 | | C_10_H_14_ | | 19.05±0.32 | | **23.9±0.29** | | 4.47±0.06 | | **29±0.15** | | 0.67±0.002 | | 10±0.08 | | 134 | |  |
| beta-Phellandrene | 1025 | | C_10_H_16_ | | 2.92±0 | | 7.02±0.08 | |  | | 6.28±0.02 | |  | |  | | 136 | |  |
| Cineole <1,8> | | 1026 | | C_10_H_18_O | | 0.56±0.02 | | 1.2±0.04 | | 9.1±0.04 | | 0.93±0.01 | | 21.3±0.39 | | **55.9±0.22** | | 154 | |
| gamma-Terpinene | | 1054 | | C_10_H_16_ | | 1.1±0.03 | | 0.43±0.01 | | 3.2±0.05 | | 0.36±0.01 | | 0.73±0.02 | | 0.34±0 | | 136 | |
| cis-Sabinene hydrate | | 1065 | | C_10_H_18_O | | 2.36±0.03 | |  | |  | | 0.7±0.03 | |  | |  | | 154 | |
| Linalool | | 1098 | | C_10_H_18_O | | 0.77±0.06 | | 1.01±0 | |  | | 0.82±0.01 | |  | |  | | 154 | |
| trans-Sabinene hydrate | | 1098 | | C_10_H_18_O | | 0.81±0.02 | |  | |  | | 0.91±0.01 | |  | |  | | 154 | |
| Fenchol <endo-> | | 1114 | | C_10_H_18_O | |  | |  | | 0.83±0.01 | |  | | 0.13±0.02 | |  | | 154 | |
| Campholenal <α-> | | 1122 | | C_10_H_16_O | |  | |  | | 0.40±0.02 | |  | | 0.18±0 | | 0.22±0 | | 152 | |
| trans-Sabinol | | 1137 | | C_10_H_16_O | |  | |  | |  | |  | | 2.56±0.01 | | 0.25±0.01 | | 152 | |
| Citronellal | | 1148 | | C_10_H_18_O | |  | | 6.53±0.04 | |  | |  | |  | |  | | 154 | |
| Isoborneol | | 1155 | | C_10_H_18_O | |  | | 0.26±0.02 | |  | |  | |  | |  | | 154 | |
| Pinocarvone | | 1160 | | C_10_H_14_O | |  | |  | | 0.19±0 | |  | |  | |  | | 150 | |
| Borneol | | 1165 | | C_10_H_18_O | |  | |  | | 2.48±0.02 | |  | | 0.24±0 | | 0.25±0.01 | | 154 | |
| Terpinen-4-ol | | 1174 | | C_10_H_18_O | | 5.06±0.02 | | 6.9±0 | |  | | 5.85±0.02 | | 0.15±0 | | 2.16±0.01 | | 154 | |
| Cryptone | | 1183 | | C_9_H_14_O | | 6.38±0.14 | | 13.8±0.02 | | 0.54±0.03 | | 14.00±0.09 | |  | |  | | 138 | |
| p-Menth-1-en-3-ol, cis-(-)- | | 1193 | | C_10_H_18_O | | 0.16±0 | | 0.27±0.01 | |  | |  | |  | |  | | 154 | |
| Estragole | | 1195 | | C_10_H_12_O | |  | |  | |  | | 0.63±0 | |  | |  | | 148 | |
| p-Menth-1-en-3-ol, trans-(-)- | | 1205 | | C_10_H_18_O | | 0.31±0 | |  | |  | | 0.62±0.02 | |  | |  | | 154 | |
| trans-Carveol | 1215 | | C_10_H_16_O | |  | |  | | 0.31±0.04 | |  | |  | |  | | 152 | |  |
| Sabinene hydrate acetate <cis-> | 1219 | | C_12_H_20_O_2_ | |  | |  | |  | | 0.6±0 | |  | | 0.14±0.02 | | 196 | |  |
| m-Cumenol | 1224 | | C_9_H_12_O | | 0.17±0.01 | | 0.29±0 | |  | | 0.32±0.01 | |  | |  | | 136 | |  |
| Cumin aldehyde | 1238 | | C_10_H_12_O | |  | | 3.69±0.06 | |  | | 3.56±0.01 | |  | |  | | 148 | |  |
| Piperitone | 1249 | | C_10_H_16_O | | 0.18±0 | | 0.24±0.01 | | 0.38±0.06 | | 0.21±0.01 | |  | |  | | 152 | |  |
| p-Cymen-7-ol | 1289 | | C_10_H_14_O | |  | | 0.96±0.02 | |  | |  | |  | |  | | 150 | |  |
| Isothymol |  | | C_10_H_14_O | | 0.47±0.02 | | 1.01±0.01 | |  | |  | |  | |  | | 150 | |  |
| Carvacrol | 1298 | | C_10_H_14_O | | 0.55±0.02 | | 1.91±0.02 | |  | | 13.2±0.02 | |  | | 0.19±0.01 | | 150 | |  |
| Isothujyl acetate | 1298 | | C_12_H_20_O_2_ | | 3.2±0.05 | |  | |  | |  | |  | |  | | 196 | |  |
| gamma-Terpinyl acetate | 1316 | | C_12_H_20_O_2_ | | 0.68±0.02 | |  | | 1.13±0 | |  | |  | |  | | 196 | |  |
| alpha-Terpinyl acetate | 1346 | | C_12_H_20_O_2_ | | 1.17±0.01 | | 2.14±0.02 | | 6.94±0.13 | |  | | 0.22±0.003 | | 0.54±0.01 | | 196 | |  |
| Citronellyl acetate | 1350 | | C_12_H_22_O_2_ | |  | |  | | 0.36±0.04 | |  | |  | |  | | 198 | |  |
| Ylangene <α-> | 1373 | | C_15_H_24_ | | 0.22±0.01 | |  | |  | |  | |  | |  | | 204 | |  |
| Epiglobulol |  | | C_15_H_26_O | | 0.28±0 | | 0.57±0 | | 1.76±0.03 | | 0.58±0.01 | | 0.22±0 | | 0.12±0.01 | | 222 | |  |
| (-)-Globulol | 1590 | | C_15_H_24_O | | 0.93±0 | | 0.81±0.04 | | 5.91±0.9 | | 0.55±0.05 | | 1.25±0.05 | | 4.61±0.15 | | 220 | |  |
| cis-Thujopsene | 1429 | | C_15_H_24_ | | 0.81±0.07 | | 0.28±0.03 | |  | | 0.11±0 | |  | | 0.16±0 | | 204 | |  |
| (E)-Thujopsene | 1431 | | C_15_H_24_ | | 0.13±0.02 | |  | |  | | 0.11±0 | |  | | 1.92±0.04 | | 204 | |  |
| Aromadendrene | 1439 | | C_15_H_24_ | |  | |  | |  | | 0.13±0 | |  | |  | | 204 | |  |
| trans-Nerolidol | 1561 | | C_15_H_26_O | |  | | 0.19±0.01 | |  | |  | |  | |  | | 222 | |  |
| Spathulenol | 1577 | | C_15_H_24_O | | **38.9±0.68** | | 11.3±0.12 | |  | | 14.3±0.14 | | 3.53±0.02 | |  | | 220 | |  |
| Caryophyllene oxide | 1582 | | C_15_H_24_O | | 0.64±0.11 | |  | |  | |  | |  | | 0.19±0.02 | | 220 | |  |
| Viridiflorol | 1592 | | C_15_H_26_O | | 0.5±0.01 | |  | | 1.43±0.01 | |  | | 0.55±0.01 | | 2.71±0.05 | | 222 | |  |
| Isospathulenol | 1628 | | C_15_H_24_O | | 0.12±0.01 | |  | |  | |  | |  | |  | | 220 | |  |
| gamma-eudesmol | 1630 | | C_15_H_26_O | |  | |  | |  | |  | | 10.2±0.11 | |  | | 222 | |  |
| Selina-3,11-dien-6α-ol | 1642 | | C_15_H_24_O | | 1.23±0.01 | |  | |  | |  | |  | |  | | 220 | |  |
| alpha-Cadinol | 1652 | | C_15_H_26_O | | 0.25±0.01 | |  | |  | |  | |  | |  | | 222 | |  |
| Torquatone | 1791 | | C_16_H_24_O | |  | |  | |  | |  | | **30±0.54** | |  | | 280 | |  |
| **Total mono.** | | | | | 25.3 | | 5.4 | | 64.2 | | 39.8 | | 21.755 | | 25.245 | |  | |  |
| **Total ox. Mono.** | | | | | 22.8 | | 37.7 | | 22.6 | | 42 | | 24.74 | | 59.405 | |  | |  |
| **Total sesq.** | | | | | 2.38 | | 0.28 | | 0 | | 0.24 | | 0 | | 2.195 | |  | |  |
| **Total ox. Sesq.** | | | | | 41.6 | | 12.9 | | 9.1 | | 15.6 | | 45.68 | | 7.51 | |  | |  |
| **Mean% identified** | | | | | 92.1 | | 96.5 | | 95.9 | | 97.96 | | 92.2 | | 94.4 | |  | |  |

Mono.: monoterpene hydrocarbons; Ox. Sesq.: oxygenated sesquiterpenes; Ox. Mono.: oxygenated monoterpenes; Sesq.: sesquiterpene hydrocarbons.

| 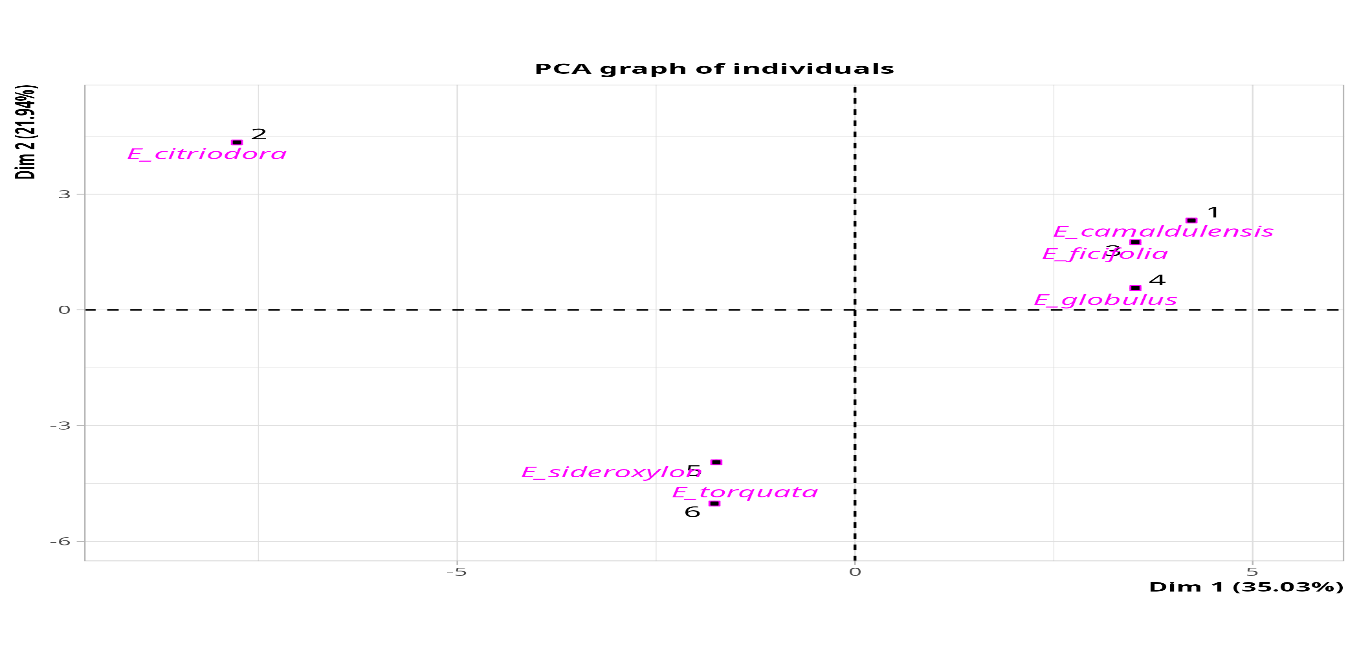 |
| --- |
| **(A)** |
| 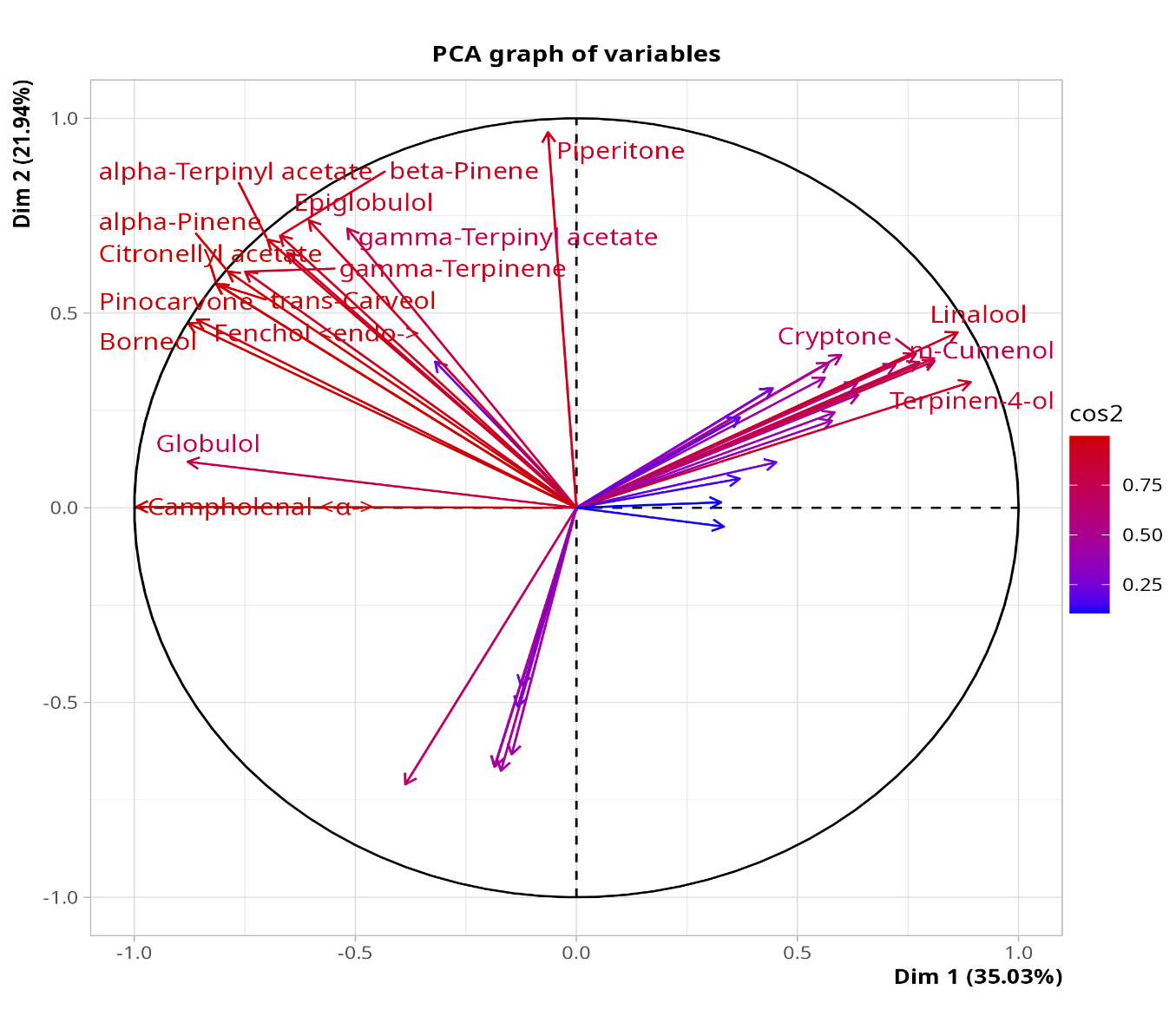 |
| **(B)** |

**Figure S2. Principal Component Analysis (PCA) of (A): different *Eucalyptus* species and (B): the main discriminatory markers**


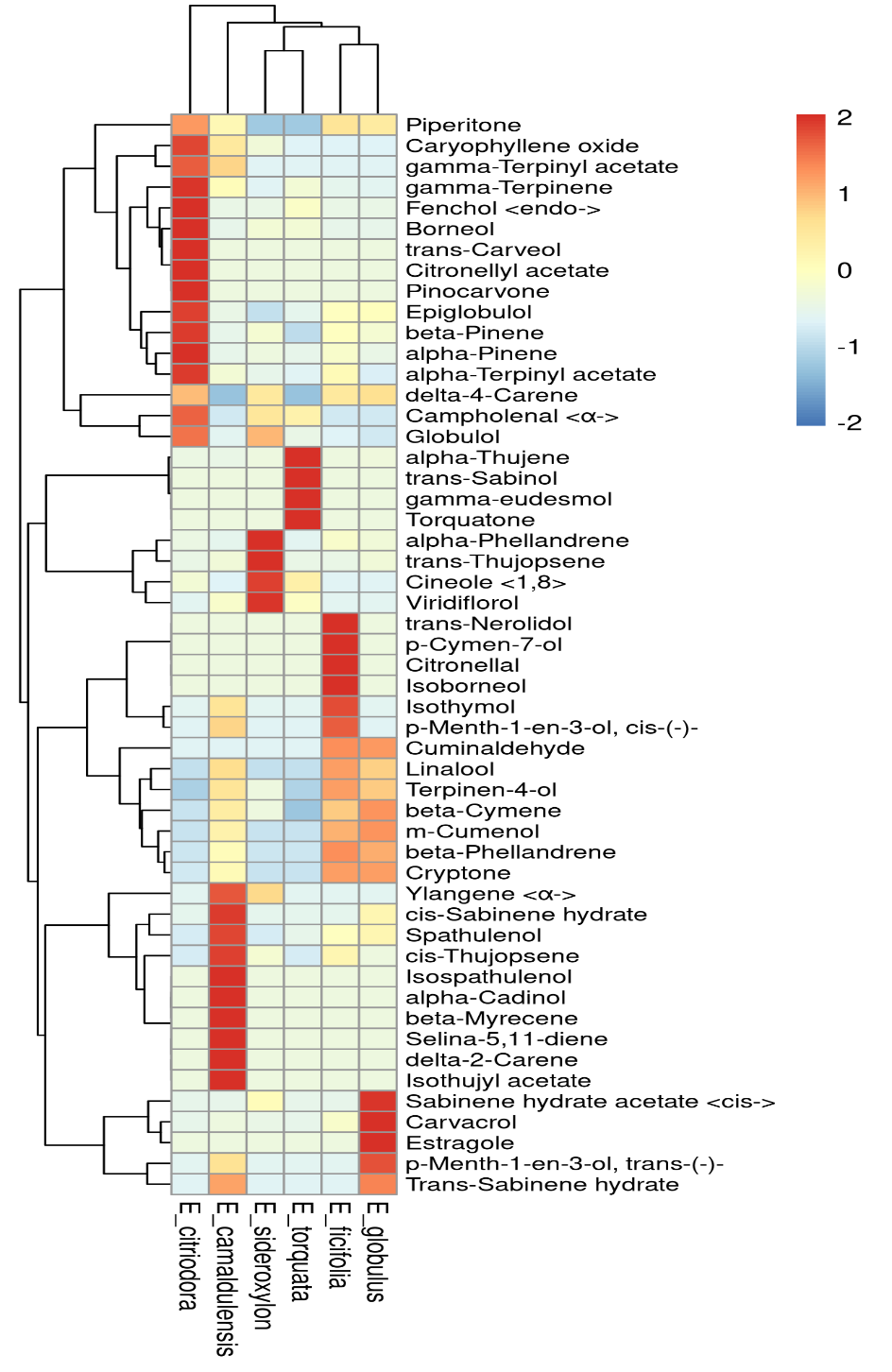


**Figure S3: Heatmap and dendrogram illustrating the different identified essential oils components levels in the six *Eucalyptus* species.**

| 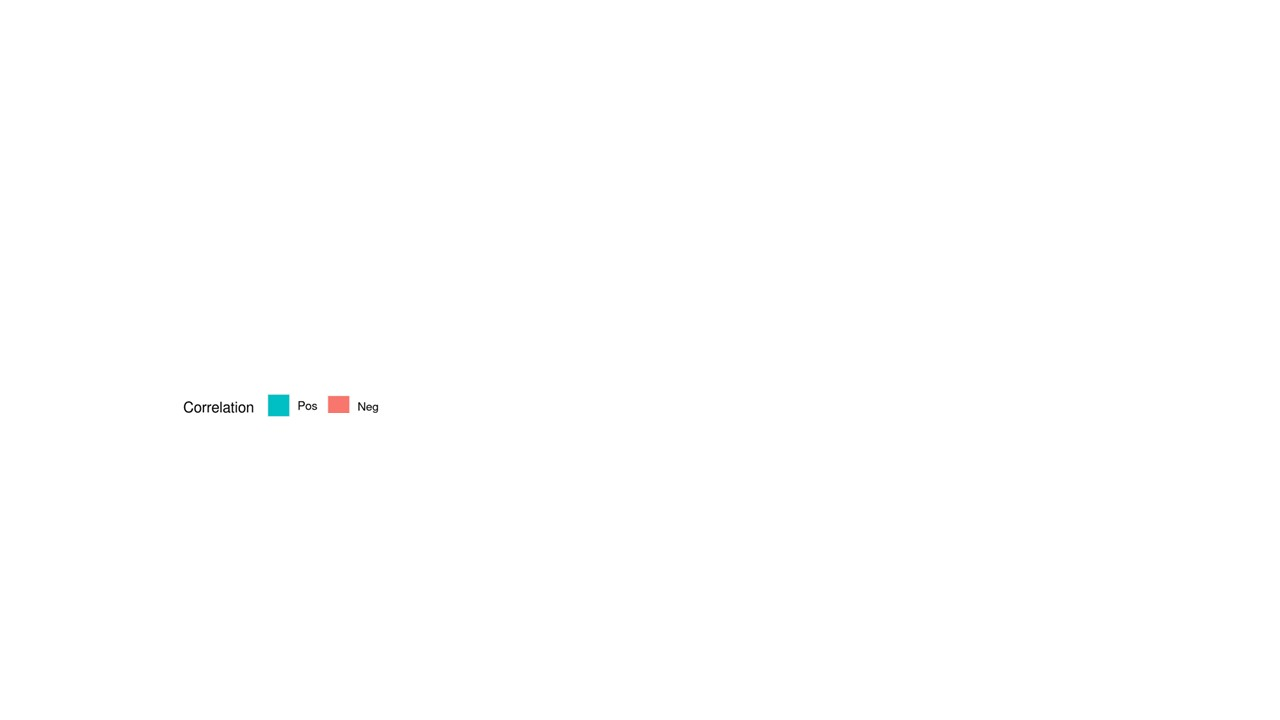  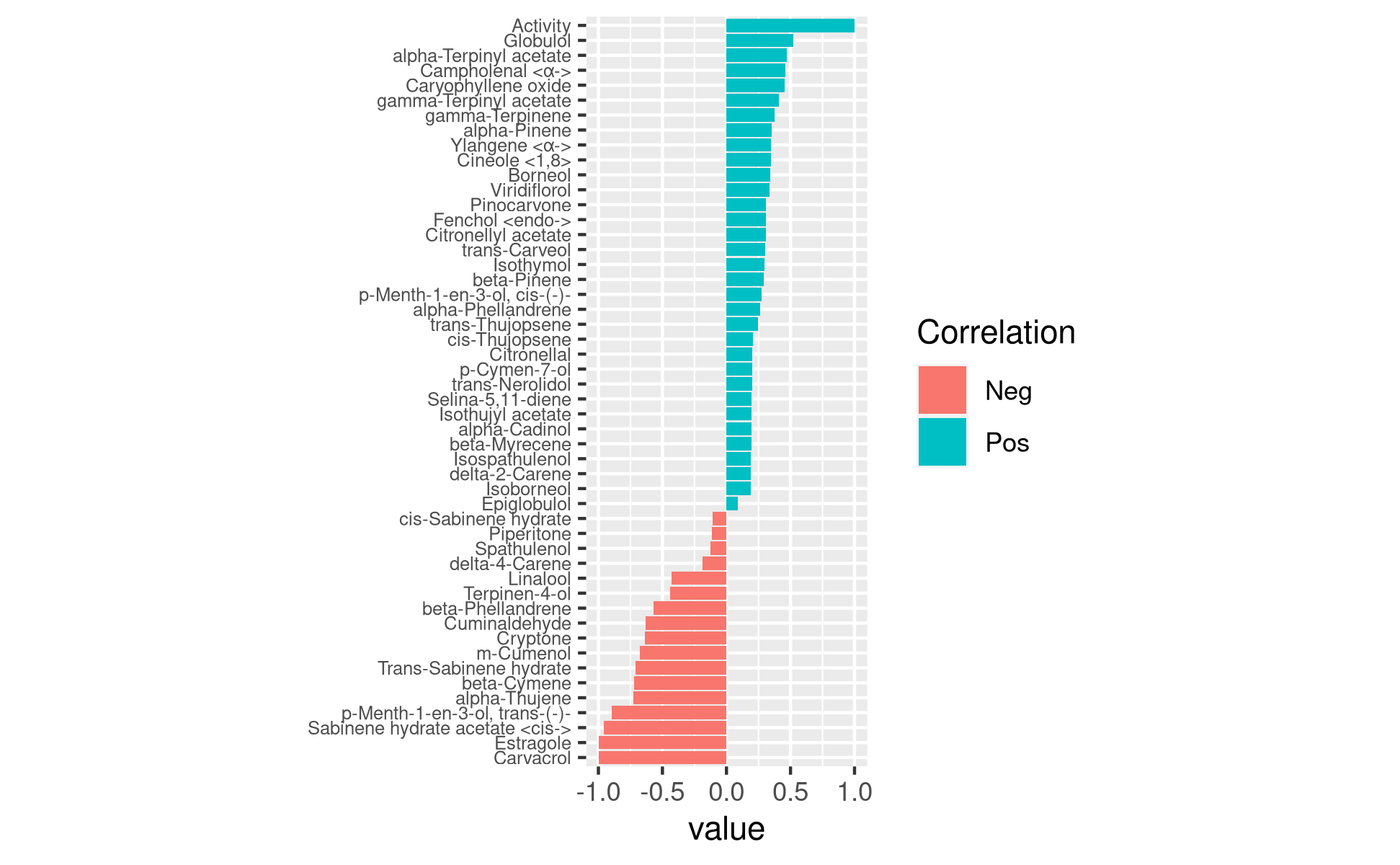 | 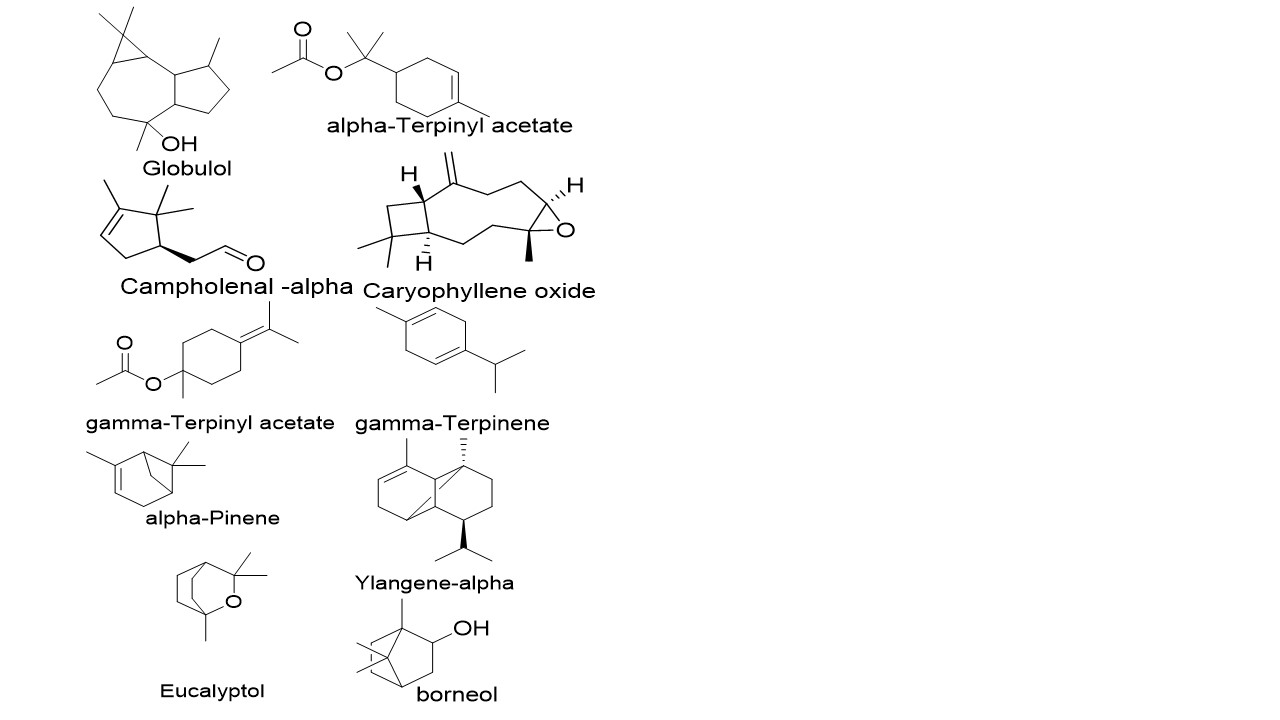 |
| --- | --- |
| **(A)** | **(B)** |

**Figure S4: Top essential oil phytochemicals correlated with *E. citriodora* anti-COVID activity (A) Pearson’s correlation coefficient indicating the relationship between each phytochemical content and the newly explored anti-COVID activity, (B) Structures of the top 10 correlated phytochemicals.**


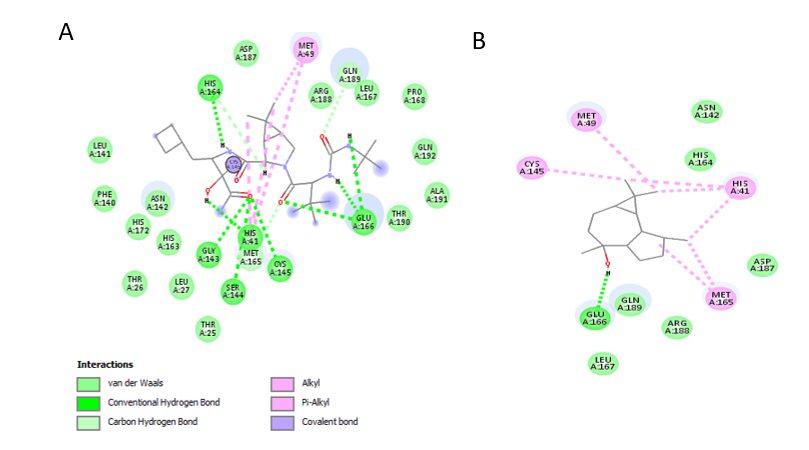


**Figure S5: The 2D binding interactions on Mpro (PDB ID: 6XQU) of A) boceprevir (E=-71.5 Kcal/mol), B) globulol (E=-26.19 Kcal/mol).**


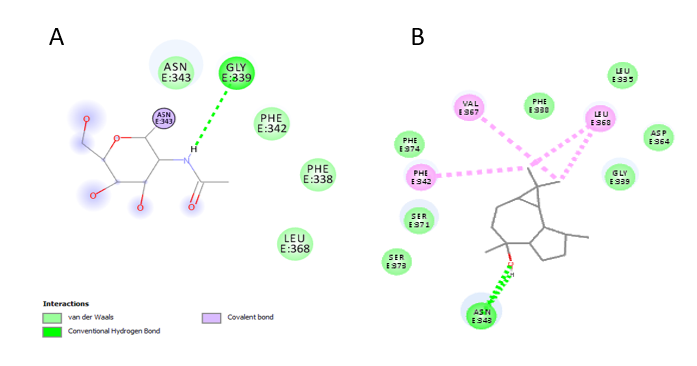


**Figure S6: The 2D binding interactions on Spike (S) protein (PDB ID: 6M0J) of A) NAG (E=-24.12 Kcal/mol) and B) globulol (E=-18.04 Kcal/mol).**


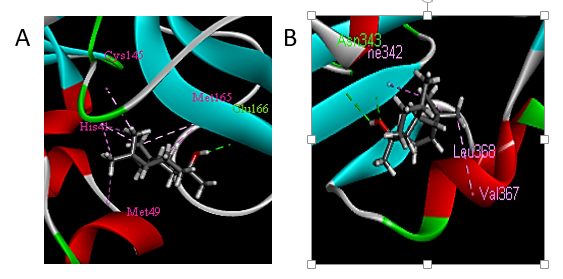


**Figure S7: The 3D binding interactions of globulol on A) Mpro (PDB ID: 6XQU) & B) Spike (S) protein (PDB ID: 6M0J).**

**Table S2. In silico ADMET prediction results for globulol**

| **Name** | **ADMET Sol level** | **ADMET A log P98** | **ADMET BBB level** | **CYP2D6 prediction**  **(Non-Inhibitor)** | **Hepatotoxic probability** | **Absorption level** | **PPB binding**  **prediction** | **Alog P98** | **PSA 2D** | **TOPKAT ames prediction** |
| --- | --- | --- | --- | --- | --- | --- | --- | --- | --- | --- |
| **Globulol** | 2 (low) | 0 | **1** | **FALSE** | TRUE | **0 (good)** | **TRUE** | 3.202 | 20.815 | **Non- Mutagen** |


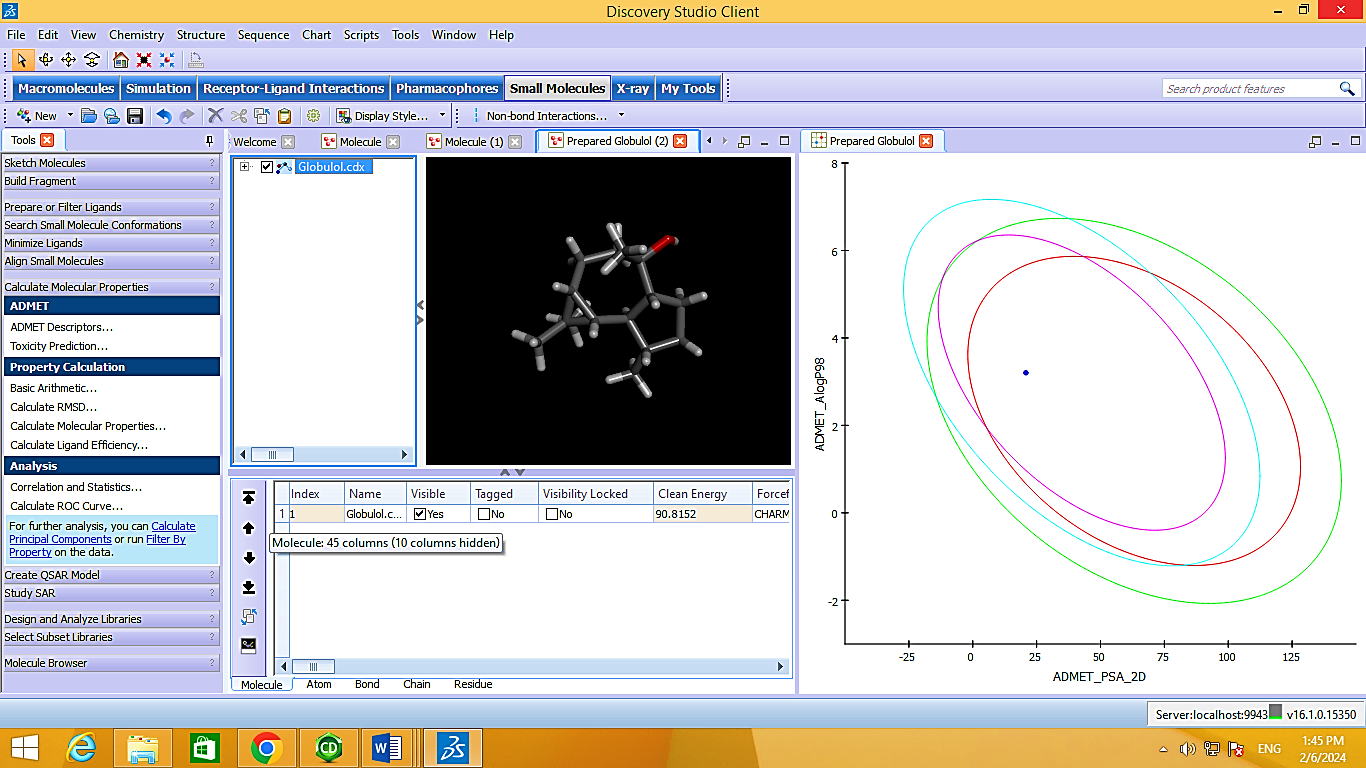


**Figure S8: The ADMET plot for globulol: calculated PSA_2D versus A log P98 properties.**

**Reference**

1. Mosmann, T., 1983 Rapid colorimetric assay for cellular growth and survival: application to proliferation and cytotoxicity assays. Journal of immunological methods. 65(1-2): p. 55-63.

2. Kandeil, A., A. Mostafa, O. Kutkat, Y. Moatasim, A.A. Al-Karmalawy, A.A. Rashad, A.E. Kayed, A.E. Kayed, R. El-Shesheny, and G. Kayali, 2021 Bioactive polyphenolic compounds showing strong antiviral activities against severe acute respiratory syndrome coronavirus 2. Pathogens. 10(6): p. 758.

3. Adams, R.P., 2007 Identification of essential oil components by gas chromatography/mass spectrometry. Vol. 456. Allured publishing corporation Carol Stream.

4. Lê, S., J. Josse, and F. Husson, 2008 FactoMineR: an R package for multivariate analysis. Journal of statistical software. 25: p. 1-18.

5. Team, R.C., 2016 R: A language and environment for statistical computing. R Foundation for Statistical Computing, Vienna, Austria. <http://www>. R-project. org/.

6. Berman, H., J. Westbrook, Z. Feng, G. Gilliland, T. Bhat, H. Weissig, I. Shindyalov, and P. Bourne, 2000 The protein data bank (www. rcsb. org). Nucleic Acids Research, 28 (1), 235-242.

7. Mohsen, A.M., Y.I. Nagy, A.M. Shehabeldine, and M.M. Okba, 2023 Thymol-Loaded Eudragit RS30D Cationic Nanoparticles-Based Hydrogels for Topical Application in Wounds: In Vitro and In Vivo Evaluation. Pharmaceutics. 15(1): p. 19.
